# Robotic “Double Loop” Roux-en-Y gastric bypass reduces the risk of postoperative internal hernias: a prospective observational study

**DOI:** 10.1007/s00464-020-07901-0

**Published:** 2020-08-28

**Authors:** Fabrizio Rebecchi, Elettra Ugliono, Silvia Palagi, Alessandro Genzone, Mauro Toppino, Mario Morino

**Affiliations:** General Surgery and Center for Minimal Invasive Surgery, Department of Surgical Sciences, University of Torino, Corso A.M. Dogliotti 14, 10126 Turin, Italy

**Keywords:** Robot, Roux-en-Y gastric bypass, RYGB, Internal hernia, Bariatric surgery

## Abstract

**Background:**

Internal herniation (IH) is a potentially serious complication after laparoscopic Roux-en-Y gastric bypass (RYGB). The aim of the study is to evaluate the incidence of IH after robot-assisted RYGB (RA-RYGB) performed with the “Double Loop” technique at our Institution.

**Methods:**

Prospective cohort study of patients submitted to RA-RYGB with the “Double Loop” technique, with a minimum follow-up of 2 years. Patients with complaints of abdominal pain at clinical visits or entering the emergency department were evaluated. Primary outcome was the incidence of IH, defined as the presence of herniated bowel through a mesenteric defect, diagnosed at imaging or at surgical exploration.

**Results:**

A total of 129 patients were included: 65 (50.4%) were primary procedures, while 64 (49.6%) were revisional operations after primary restrictive bariatric surgery. Mean age was 47.9 ± 10.2 years, mean weight, and body mass index were, respectively, 105.3 ± 22.6 kg and 39.7 ± 9.6 kg/m^2^. Postoperative morbidity rate was 7.0%. Mean follow-up was 53.2 ± 22.6 (range 24–94) months. During the follow-up period, a total of 14 (10.8%) patients entered the emergency department: 1 patient had melena, 4 renal colic, 1 acute cholecystitis, 2 gynecologic pathologies, 2 anastomotic ulcers, 1 perforated gastric ulcer, 1 diverticulitis and 2 gastroenteritis. There were no diagnoses of IH. During the follow-up period, no patient experienced recurrence of symptoms.

**Conclusions:**

In the present study, the robotic approach confirms the low complication rate and absence of IH after “Double Loop” RA-RYGB in a large case-series at a medium-term follow-up.

Laparoscopic Roux-en-Y gastric bypass (RYGB) is one of the most commonly performed bariatric procedures [1]. Several studies documented the effects of RYGB on weight loss and resolution of comorbidities at long term follow-up [2, 3].

IH is a troublesome complication after RYGB. Clinical manifestations of IH are highly variable, ranging from mild intermittent abdominal pain to life-threatening complications such as small bowel incarceration, obstruction or strangulation, with a non-negligible mortality rate of 1.6% [4, 5].

IH occurs due to the presence of inter-mesenteric spaces created after the mobilization of the Roux limb, through which the small bowel can herniate. Potential IH sites after RYGB are the space through the mesenteric defect created at the jejuno–jejunal (JJ) anastomosis site, and the space between the transverse mesocolon and the afferent limb mesentery of the gastro–jejunal (GJ) anastomosis (Petersen's space) [6]. Additionally, IH can occur at the level of the transverse mesocolon in case of retrocolic reconstruction.

The mean incidence of IH after laparoscopic RYGB is 2.5%, that is higher than reported with the open approach, probably due to the reduced adhesion formation of the laparoscopic approach [5, 7–10]. Several technical considerations, mainly involving the route of the Roux limb (antecolic Vs. retrocolic), and the closure Vs. non-closure of the mesenteric defects have been evaluated in attempt to decrease the risk of IH [11–14].

Recently, a single-incision laparoscopic surgery technique has been described by Tacchino et al. as the “Double Loop” gastric bypass [15]. This technique does not require the opening of the mesentery during the construction of the Roux limb, avoiding the risk of IH formation at this site.

The aim of this study is to describe our experience with the “Double Loop” RA-RYGB and to assess the incidence of IH in our series.

## Materials and methods

**Fig. 1:**
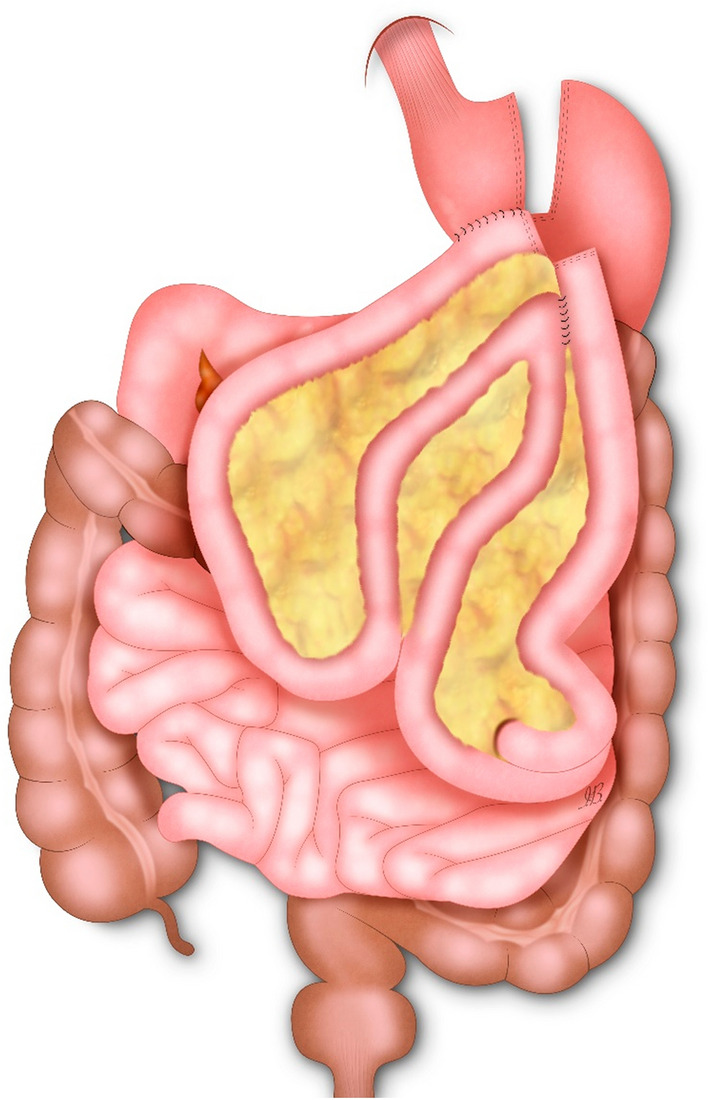
Double Loop gastric bypass

Analysis of a prospectively collected database of patients submitted to RA-RYGB with the “Double Loop” technique at our Institution (Fig. 1). The study was approved by the local Ethical Committee.

We included in the study consecutive adult patients (≥ 18 years) with indication to RA-RYGB as both primary and revisional surgery. Patients undergoing primary RA-RYGB for morbid obesity met the 1991 National Institute of Health (NIH) consensus criteria [16]. Indications to RA-RYGB as a revisional procedure after primary restrictive bariatric surgery were weight loss failure or complications of the index procedure [17].

All patients underwent a comprehensive preoperative work-up that included clinical examination, blood samples, upper endoscopy, and radiological series. *Helicobacter pylori *infection (HP) was eradicated before surgery, if present. Patients signed a detailed informed consent regarding the surgical procedure at preoperative clinical examination.

### Surgical procedure

#### Patient positioning and port placement

Patients were positioned in a 30° reversed Trendelenburg position with the lower limbs abducted and the right arm extended.

A 12-mmHg pneumoperitoneum was created with a Veress needle inserted in the left hypochondrium. The 30° optical system was introduced through a midline trocar positioned approximately halfway between the xiphoid and the umbilicus. In all patients other five trocars were positioned under vision: three robotic trocars at the same level as the camera (two on the left and one on the right), and two assistant trocars in the right flank, one through which the laparoscopic linear stapler was introduced and one for liver retraction.

#### Formation of the gastric pouch

The first step of RA-RYGB was the creation of the gastric pouch, performed laparoscopically. The stomach was dissected at the lesser gastric curvature starting at 6 cm from the esophago–gastric junction. The gastric pouch was created with multiple purple cartridges of a laparoscopic linear stapler (EndoGIA® Tristaple, Covidien, Mansfield, MA, USA) calibrated on a 12-mm orogastric bougie.

#### Gastro-jejunal anastomosis

We proceeded with the docking of the DaVinci Surgical System (Intuitive Surgical Inc, Sunnyvale, CA, USA). A loop of small bowel 100 cm from the ligament of Treitz, that could be brought upward in an antecolic antegastric fashion without tension, was identified. A robotic hand-sewn right-oriented end-to-side GJ anastomosis with a running two layers suture (Vicryl for the outer seromuscular layer and PDS for the inner mucomucosal layer) was performed.

#### Jejuno-jejunal anastomosis

Differently from the technique described by Tacchino et al., first we performed the transection of the small bowel in closed proximity to the GJ anastomosis with a linear stapler, that was inserted through a minimal passage in the mesentery adjacent to the intestinal wall [15]. This allowed to ease the creation of the JJ anastomosis without opening the mesentery and without tension.

Then we measured a second loop of small bowel 150 cm from the GJ anastomosis; we performed a side-to-side mechanical JJ anastomosis with the cut end of the previously transected small bowel, without opening the mesentery, using a linear stapler oriented from the right to the left of the patient. The enterotomies were then closed with running absorbable sutures.

**Fig. 2:**
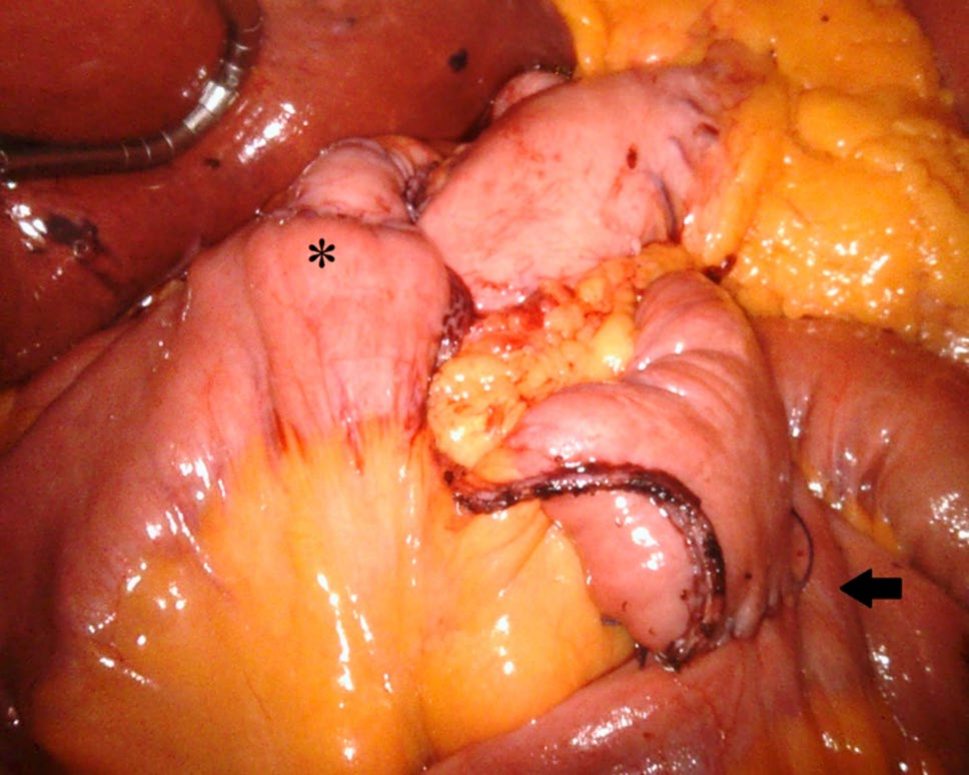
End of the procedure. Both the GJ (asterisk) and the JJ anastomoses (arrow) are in the supramesocolic compartment. The mesenteric integrity is preserved

At the end of the procedure both the GJ and the JJ anastomosis were in the supramesocolic compartment, and the integrity of the mesentery was preserved (Fig. 2).

A methylene blue test was performed to check for anastomotic leaks and patency, and a peri-anastomotic drain was placed.

### Follow-up protocol

Postoperative complications were evaluated using the Clavien–Dindo classification [18].

After discharge, patients were re-assessed in outpatient clinic at 3, 6, 12, 24 months and yearly thereafter to evaluate weight, body mass index (BMI), and percentage of excess weight loss (%EWL).

Patients with complaints of abdominal pain at clinical visit and patients presenting at the Emergency Department (ED) for acute abdominal pain or symptoms of bowel obstruction were evaluated.

All patients entering the ED were submitted to clinical examination, blood samples and the appropriate imaging according to the clinical scenario: ultrasonographic scan in case of abdominal pain; abdominal X-rays in the presence of symptoms of bowel obstruction; upper gastrointestinal endoscopy for complaints of heartburn or epigastric pain. Computed Tomography (CT) scan was performed in doubtful cases after first-level imaging.

IH was defined as the presence of herniated bowel through a mesenteric defect, diagnosed at imaging or at surgical exploration.

The statistical analysis was performed with Microsoft Excel (Microsoft Excel®, version 2001, build 12430.20184). Quantitative data are given as mean and standard deviation, and categorical data are expressed as percentage.

## Results

From January 2012 to December 2017, a total of 129 patients underwent RA-RYGB with the “Double Loop” technique at our Institution: 65 (50.4%) were primary RA-RYGB and 64 (49.6%) were revisional RA-RYGB after previous restrictive bariatric surgery: five patients have been converted from adjustable gastric banding, 17 from sleeve gastrectomy and 42 from vertical banded gastroplasty. Mean age was 47.9 ± 10.2 years, mean preoperative weight and BMI were, respectively, 105.3 ± 22.6 kg and 39.7 ± 9.6 kg/m^2^. Table 1 summarizes baseline patient characteristics.

**Table 1:** Patient baseline characteristics

Total *N* = 129		
Mean age (years)	47.9 ± 10.2	
Sex		
Male	18 (13.9%)	
Female	111 (86.1%)	
Preoperative weight (kg)	105.3 ± 22.6	
Preoperative BMI (kg/m^2^)	39.7 ± 9.6	
Comorbidities		
Hypertension	51 (39.5%)	
Diabetes	17 (13.2)	
Obstructive sleep apnoea syndrome	20 (15.5%)	
Dyslipidemia	9 (7.0%)	
Upper endoscopy		
Esophagitis	80 (62.0%)	
Barrett's esophagus	6 (4.6%)	
* Helicobacter pylori*	16 (12.4%)	
Reasons for reoperation (*n* = 64)		
Insufficient weight loss	7 (10.9%)	
Weight regain	11 (17.2%)	
Dysphagia	26 (40.7%)	
Gastro-esophageal reflux disease	20 (31.2%)	

All surgical procedures were performed by the same two experienced robotic and laparoscopic surgeons (MM, FR). All patients underwent RA-RYGB with a DaVinci Si surgical system (Intuitive Surgical Inc, Sunnyvale, CA, USA) until June 2015 (51 patients), then with a DaVinci Xi robotic platform (78 patients). Mean operative time was 241.6 ± 32.8 min. Conversion to open surgery was necessary in 2 (1.5%) cases for dense adhesions. There were no anastomotic leaks detected at methylene blue test or other intraoperative complications. Postoperative morbidity rate was 7.0%: 8 grade II (two bleeding requiring blood transfusions, five pneumonia, and one splenic infarction) and one grade IIIB (small bowel perforation requiring reintervention) postoperative Clavien–Dindo complications. There were no deaths. Mean hospital stay was 5.3 ± 3.3 days.

**Fig. 3:**
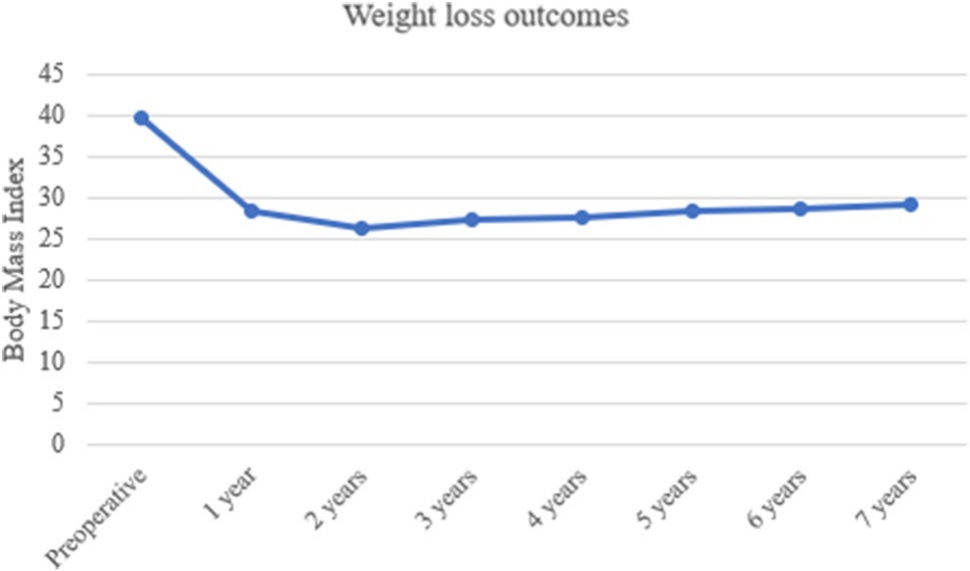
Weight loss outcomes

Mean follow-up was 53.2 ± 22.6 (range 24–94) months. Three (2.3%) patients were lost to follow-up. Two (1.5%) patients died for unrelated causes: one for severe pneumonia 2 years after surgery and one for metastatic breast cancer approximately 3 years after RYGB. Weight loss data up to 7 years of follow-up are shown in Fig. 3. No patient developed symptomatic IH during the study period.

A total of 14 (10.8%) patients entered the ED during the follow-up period for abdominal pain or symptoms of bowel obstruction. One patient had an episode of melena that did not require therapy one month after surgery, four had ultrasonographic diagnosis of renal colic, one patient was diagnosed with acute cholecystitis and underwent laparoscopic cholecystectomy, while two patients were submitted to laparoscopic hysterectomy for gynecologic issues. Two patients with severe heartburn had endoscopic evidence of esophagitis and anastomotic ulcers that resolved with proton pump inhibitors treatment. Four patients with fever and increased inflammation markers underwent CT scan: one patient had acute diverticulitis, two patients had negative CT scan and were diagnosed with acute gastroenteritis, respectively, 6 months and 2 years after RA-RYGB, while one patient underwent surgical re-exploration and repair of a perforated gastric ulcer 3 years after surgery. The postoperative course was uneventful. During the follow-up period no recurrence of symptoms was observed.

Furthermore, a total of four patients with complaints of biliary colic during outpatient clinical visits underwent ultrasonic examination with evidence of cholelithiasis and were submitted to elective laparoscopic cholecystectomy. In all patients undergoing surgical exploration during the follow-up period, no signs of IH were detected. Furthermore, a total of five pregnancies occurred without complications.

## Discussion

IH is one of the most common causes of small bowel obstruction after RYGB [19–21]. The incidence of IH is reported as high as 14% and seems to be highest during the first 2 years after surgery, corresponding with the greatest weight loss [4, 5, 8]. Several technical aspects, mainly involving the route of the Roux limb and the method of mesenteric defects closure, have been investigated in order to identify factors that might prevent the occurrence of IH after RYGB.

The antecolic antegastric is the most commonly performed approach [22]. It reduces the rate of IH eliminating one of the three possible hernia site that are created with the retrocolic construction, the transmesocolic defect. Several meta-analyses showed a reduced rate of IH with the antecolic route compared to the retrocolic retrogastric GJ reconstruction [9, 11, 12]. However, the antecolic approach could lead to increased tension on the GJ anastomosis in patients with unfavorable anatomy [5].

Closure of the mesenteric defects, eliminating potential sites of herniation, was advocated to prevent IH formation. Various techniques of closure have been described, including different types of suture (either with absorbable or non-absorbable material), the use of staplers and of fibrin sealant reinforcements [10, 23–25]. Several authors reported a decreased rate of IH with mesenteric defects closure [5, 13, 14, 26, 27]. On the other hand, closure of the mesenteric defects was associated with higher risk of complications such as bleeding due to injury of the mesenteric vessels, and kinking of the anastomosis leading to small bowel obstruction [13, 28, 29].

Because of the limited evidence, consisting of low quality and small heterogeneous studies with short follow-up, no definitive conclusion can be drawn.

The “Double Loop” gastric bypass technique was first described by Tacchino in 2010 as a single-incision laparoscopic surgery approach on two morbidly obese patients [15]. The novelty of this procedure was the avoidance of mesenteric opening during the construction of the Roux limb; therefore, the anastomoses were closed to each other and were both in the supramesocolic compartment. However, postoperative follow-up data and rate of IH were not reported as they were not an endpoint of the study.

This technique was tested also by Palmisano et al. in 2014 with standard laparoscopic approach [30]. The authors reviewed 44 patients submitted to this technique, with a mean follow-up of 18 months. They reported a mean operative time of 157 ± 12 min. Anastomotic complications occurred in 22.7% of patients: seven leaks of the GJ anastomosis detected intraoperatively and repaired with interrupted stitches, one leak of the JJ requiring reintervention on postoperative day 1, and 2 GJ strictures requiring endoscopic dilatation. No IH were diagnosed during their 18-month follow-up.

Our study is the first to describe the results of the “Double Loop” RA-RYGB in a large case-series, focusing on the rate of IH, after an adequate follow-up period. The zero incidence of IH with this technique could be explained by the construction of an antecolic, antegastric right-oriented GJ anastomosis and by the absence of potential weakness points in the mesentery through which the small bowel could herniate. With this technique, the transection of the small bowel without opening the mesentery, fixes the GJ and the JJ anastomosis close to each other in the supramesocolic compartment; in this way, the mesentery integrity is preserved. In our opinion, the separation of the two anastomoses during conventional laparoscopic RYGB generates tension on the transected mesentery, that acts as a stiff border through which the small bowel can herniate and get incarcerated. After “Double Loop” gastric bypass two spaces are created due to the upward shifting of the small bowel loop used to create the two anastomoses: one between the afferent limb and the stomach posteriorly, at the site of the GJ anastomosis, and the second one between the efferent limb and the small bowel loop at the site of the JJ anastomosis. However, these are virtual spaces with no rigid boundaries, and for anatomical reasons is high unlikely that an IH could enter and get incarcerated.

The two years minimum follow-up of our study, that corresponds with the greatest weight loss, should be sufficient to diagnose the majority of IH. However, the risk of developing IH is theoretically lifelong and longer follow-up could reveal IH not yet detected.

## Conclusions

This study demonstrates that the “Double Loop” RA-RYGB is safe and feasible, with low rates of complications. This technique is associated with no incidence of IH in our series at a medium-term follow-up. Further studies with longer follow-up are necessary to validate our results.
